# Biologics and cardiovascular events in inflammatory arthritis: a prospective national cohort study

**DOI:** 10.1186/s13075-018-1669-x

**Published:** 2018-08-07

**Authors:** Joshua L. Lee, Premarani Sinnathurai, Rachelle Buchbinder, Catherine Hill, Marissa Lassere, Lyn March

**Affiliations:** 1 Sydney Medical School, University of Sydney, Sydney, Australia; 2 Institute of Bone and Joint Research, Kolling Institute, Northern Sydney Local Health District, St Leonards, NSW Australia; 3 Department of Rheumatology, Royal North Shore Hospital, Reserve Road, St Leonards, NSW 2065 Australia; 4 Monash Department of Clinical Epidemiology, Cabrini Institute, Melbourne, VIC Australia; 5 Centre of Cardiovascular Research & Education in Therapeutics, School of Public Health and Preventive Medicine, Monash University, Melbourne, VIC Australia; 6 Department of Rheumatology, The Queen Elizabeth Hospital, Adelaide, SA Australia; 7 University of Adelaide, Adelaide, SA Australia; 8 School of Public Health and Community Medicine, University of New South Wales, Sydney, NSW Australia; 9 Rheumatology Department, St George Hospital, Sydney, NSW Australia

**Keywords:** Biologicals, Cardiovascular disease, Rheumatoid arthritis, Psoriatic arthritis, Ankylosing spondylitis

## Abstract

**Background:**

Inflammatory arthritides including rheumatoid arthritis (RA), psoriatic arthritis (PsA) and ankylosing spondylitis (AS) are associated with increased risk of cardiovascular disease. This process may be driven by systemic inflammation, and the use of tumour necrosis factor (TNF) inhibitors could therefore potentially reduce cardiovascular risk by reducing this inflammatory burden. The aims of this study were to evaluate whether the risk of cardiovascular events (CVEs) in patients with inflammatory arthritis is associated with treatment with anti-TNF therapy, compared with other biologics or non-biologic therapy, and to compare the CVE risk between participants with RA, PsA and AS.

**Methods:**

Data from consecutive participants in the Australian Rheumatology Association Database with RA, PsA and AS from September 2001 to January 2015 were included in the study. The Cox proportional hazards model using the counting process with time-varying covariates tested for risk of having CVEs, defined as angina, myocardial infarction, coronary artery bypass graft, percutaneous coronary intervention, other heart disease, stroke/transient ischaemic attack or death from cardiovascular causes. The model was adjusted for age, sex, diagnosis, methotrexate use, prednisone use, non-steroidal anti-inflammatory use, smoking, alcohol consumption, hypertension, hyperlipidaemia, diabetes and functional status (Health Assessment Questionnaire Disability Score).

**Results:**

There were 4140 patients included in the analysis, totalling 19,627 patient-years. After multivariate adjustment, the CVE risk was reduced with anti-TNF use (HR 0.85, 95% CI 0.76–0.95) or other biologic therapies (HR 0.81, 95% CI 0.70–0.95), but not in those who had ceased biologic therapy (HR 0.96, 95% CI 0.83–1.11). After adjustment, no significant difference in CVE risk was observed between participants with RA and PsA (HR 0.92, 95% CI 0.77–1.10) or AS (HR 1.14, 95% CI 0.96–1.36).

**Conclusions:**

Current biologic use was associated with a reduction in major CVEs. No reduction in CVE risk was seen in those who had ceased biologic therapy. After adjustment, the CVE risk was not significantly different between RA, AS or PsA.

## Background

Inflammatory arthritides such as rheumatoid arthritis (RA), ankylosing spondylitis (AS) and psoriatic arthritis (PsA) impose a heavy burden of morbidity and mortality on populations worldwide. A significant component of this is the two-fold increased risk of cardiovascular events (CVEs) [1], with some evidence for increasing risk with longer disease duration [2–4]. It has been proposed that this is due to inflammatory processes driven by cytokines such as tumour necrosis factor (TNF), with a high inflammatory burden driving autoantibody production and apoptosis of endothelial cells to cause vascular damage [5] and a pro-thrombotic state [6].

The use of TNF inhibitors could therefore potentially reduce cardiovascular risk by controlling systemic inflammation. A recent study demonstrated that an RA cohort with disease onset after the year 2000 did not have an increased mortality risk compared to the general population, whereas those with disease onset prior to 2000 were at increased risk [7]. Several studies have demonstrated that treatment of inflammatory arthritis with TNF inhibitors is associated with an improvement in surrogate markers of cardiovascular health such as endothelial stiffness, biochemical lipid profile and carotid intima-media thickness [8–13].

There is conflicting evidence regarding clinical cardiovascular endpoints such as rate of myocardial infarction, stroke and cardiovascular-related death after treatment with biologics in patients with RA. Some studies report a lower risk of CVEs [14, 15], while others report no significant difference [16, 17]. Studies assessing cardiovascular risk in RA have been performed in locations including North America [14, 18, 19], Britain [20] and Sweden [21], but as yet no studies have been undertaken in the Australian context where there are stringent criteria for accessing biologic therapy. Furthermore, little research has been done to establish the effect of biologics on the CVE rate for inflammatory arthritis apart from RA. Thus, wider research is warranted in a range of arthritic conditions to examine whether biologic therapy is helpful beyond direct arthritic control in these patients.

The aim of this study was to determine whether the risk of CVEs in patients with RA, AS or PsA was associated with treatment with anti-TNF therapy, compared with other biologics or non-biologic therapy, and to compare the CVE risk between arthritis diagnoses.

## Methods

The Australian Rheumatology Association Database (ARAD) is a national voluntary registry for patients with inflammatory arthritis (RA, AS, PsA and juvenile idiopathic arthritis). Details regarding the ARAD methodology have been described previously [22]. Briefly, participants with inflammatory arthritis complete self-reported questionnaires in paper or online format. Initially, these were completed biannually; however, from January 2014, the frequency of questionnaires was decreased to annually after the first 2 years of follow-up. The participant questionnaires include self-reported demographic details, current and past use of medications for arthritis, and current and past co-morbid medical conditions. Participants also complete patient-reported outcome measures including the Health Assessment Questionnaire Disability Score (HAQ)—a measure of functional status with scores ranging from 0 to 3 where higher scores indicate greater disability [23].

The majority of participants are referred by their treating rheumatologist (98.5%) and a small proportion is self-referred. Rheumatologists complete basic information at baseline including demographics and diagnosis. Cause of death is validated by data linkage to the Australian National Death Index, which provides verified International Statistical Classification of Diseases and Related Health Problems, 10th revision (ICD-10) coding for cause of death [24]. Skilled data-entry personnel review inputs and correct errors, contacting participants for clarification as required.

Consecutive participants with RA, PsA or AS who had completed at least two separate ARAD questionnaires from database inception on 12 September 2001 to 28 January 2015 were included in the analysis. Demographic details, diagnosis, date of questionnaire, medications, medical history, HAQ score and, when applicable, cause of death were extracted from the ARAD on 28 January 2015. The primary outcome of interest was the composite rate of CVEs. CVEs were defined as any stable/unstable angina, myocardial infarction, coronary artery bypass graft, percutaneous coronary intervention, other heart disease (e.g. valvular), stroke/transient ischaemic attack or death from cardiovascular causes. This was in line with definitions commonly used in the literature [25–28]. Identification of CVEs, other than cardiovascular-related death, was based upon participants’ self-report. Based on ICD-10 codes obtained via data linkage with the Australian National Death Index, any cause of death in Chapter IX (Blocks I00–I99, “Diseases of the circulatory system”) was identified as a cardiovascular-related death and included in the composite measure of CVEs [24, 29].

### Statistical methods

Survival analysis was conducted in SAS 9.4 using the Cox proportional hazards model and the counting process method to estimate hazard ratios (HRs) and corresponding 95% confidence intervals (95% CIs) for the rate of CVEs in patients who had anti-TNF biologic treatment, as compared to those with other biologic therapy or no biologic therapy. A repeated-events counting process model was utilised rather than a time-to-first-event model in order to account for the increased risk from multiple events during follow-up [30–32]. Participants who did not experience any CVE were right censored at the end of follow-up.

The main predictor of interest was biologic therapy use. The ARAD codes individual biologic therapies as current, previous, never or unknown, at each reported time point. For this analysis, biologic therapies were coded by conflation into anti-TNF (infliximab, etanercept, adalimumab, golimumab, certolizumab pegol) or other (anakinra, rituximab, abatacept, tocilizumab) to form the mutually exclusive groups of current anti-TNF use, current other biologic use, previous biologic use (any) or biologic-naïve. Data points where participants reported unknown biologic use were treated as missing and were excluded from the analysis. Medication use was coded as a time-varying variable to account for participants being put on different treatment across the longitudinal cohort study. Included participants were assumed to continue their reported biologic therapy for the interval between surveys.

Other participant characteristics included in the model as explanatory variables were age, sex, arthritis diagnosis, disease duration, alcohol usage and smoking status. Treatment status for non-steroidal anti-inflammatory drugs (NSAIDs), methotrexate and prednisone/prednisolone was coded as never, current, past or unknown. Co-morbid medical illnesses which are known cardiac risk factors (hyperlipidaemia, hypertension or diabetes) were also included as explanatory variables. These were self-reported in the ARAD as current, past, never or unknown. Those that were reported as current or past were coded as a positive history while those reported as never were coded as negative. Data points where participants reported an unknown history were treated as missing data and dropped from the analysis. The HAQ was included as a continuous variable. Univariate analyses were conducted and continuous variables were checked for linearity. Variables with a *p* value less than 0.25 in the univariate analysis were included in the multivariate model. Multi-collinearity in the multivariate model was evaluated using variance inflation factors (VIFs).

Multivariate analysis was performed using the backwards elimination method and the χ^2^ likelihood ratio test. Hazard ratios (HRs) with 95% confidence intervals (95% CIs) were reported using an α value of 0.05. The risk of CVEs was compared between RA, AS and PsA using the HR for each diagnosis from the final adjusted multivariate model. The results were reported in accordance with the “Strengthening the Reporting of Observational Studies in Epidemiology” (STROBE) guidelines [33].

## Results

**Fig. 1:**
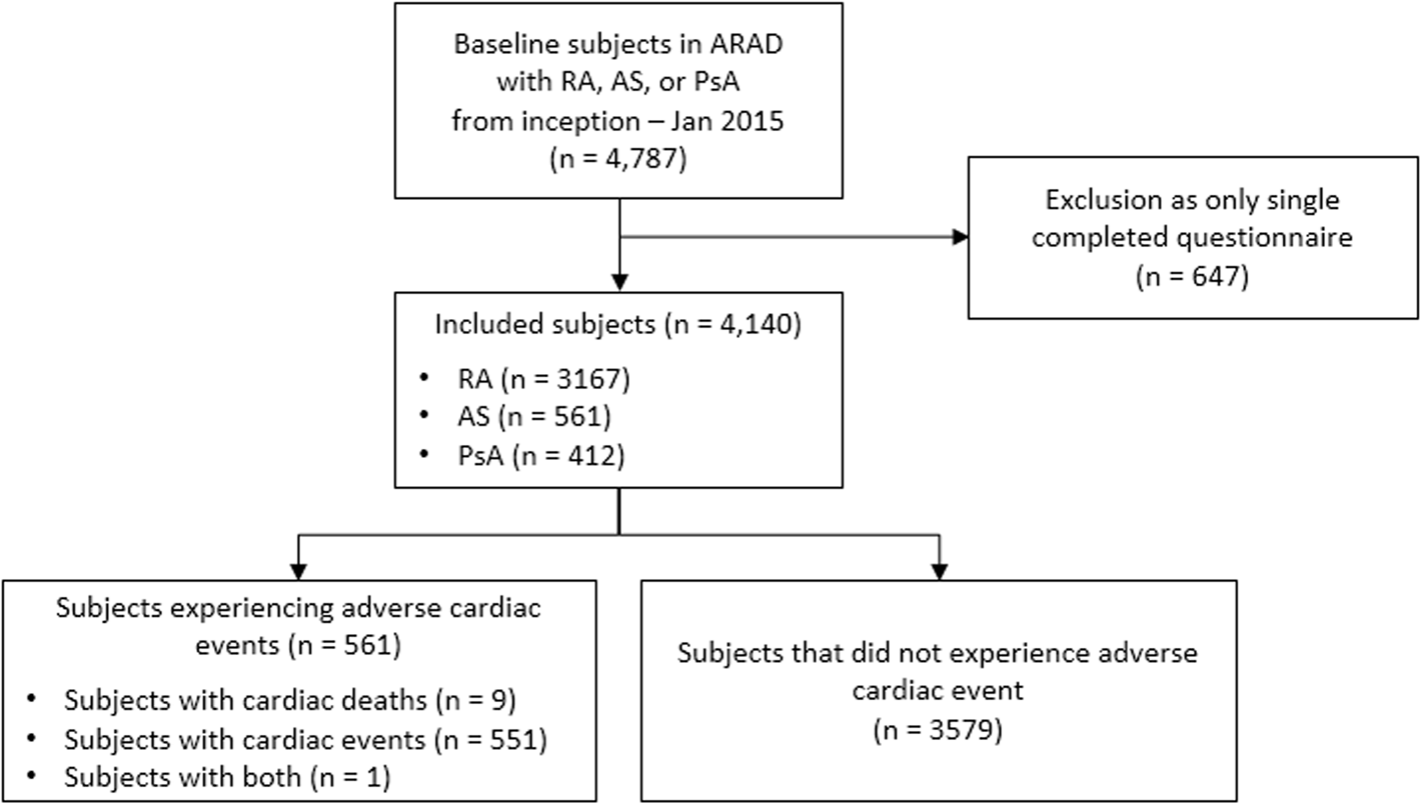
Flow diagram for participant inclusion from the ARAD. ARAD Australian Rheumatology Association Database, AS ankylosing spondylitis, PsA psoriatic arthritis, RA rheumatoid arthritis

Between 2001 and 2015, there were 4787 participants enrolled in the ARAD with a diagnosis of RA, AS or PsA (Fig. 1). Participants with only a single completed questionnaire (*n* = 647) were excluded. Thus, 4140 participants were included in the analysis, totalling 32,844 completed questionnaires. Participant demographics at the time of enrolment in the ARAD are presented in Table 1. The median age was 56 years (interquartile range (IQR) 46–64 years), and 33.6% were male. The majority of participants had a diagnosis of RA (*n* = 3167, 76.5%), 561 (13.6%) had AS and 412 (10.0%) had PsA. The median time since diagnosis was 10 years (IQR 4–19 years) and the median (IQR) HAQ was 1.13 (0.50–1.75). Participants who had ever smoked regularly comprised 37.2% of the sample. In terms of alcohol use, 13.2% of participants were daily users, 54.4% occasional users and 32.4% non-users. Self-reported co-morbidities included hypertension (34.9%), hyperlipidaemia (19.1%) and diabetes (7.6%).

**Table 1:** Patient characteristics at ARAD enrolment (*n* = 4140)

	Median (interquartile range)	Number	Percentage
Age (years)	56 (46–64)		
< 40		569	13.7
40–49		779	18.8
50–59		1182	28.6
60–69		1067	25.8
≥ 70		543	13.1
Sex			
Male		1393	33.6
Female		2747	66.4
Disease duration (years)	10 (4–19)		
≤ 5		1075	26.0
6–10		833	20.1
11–20		694	16.8
21–30		536	12.9
> 30		986	23.8
Unknown^a^		16	0.4
Diagnosis			
RA		3167	76.5
AS		561	13.5
PsA		412	10.0
Health Assessment Questionnaire Disability Score^b^	1.13 (0.50–1.75)		
Smoking regularly			
Current or past		1540	37.2
Never		2129	51.4
Unknown^a^		471	11.4
Alcohol consumption			
Never		1342	32.4
Sometimes		2253	54.4
Every day		545	13.2
History of co-morbid medical conditions			
Diabetes			
No		3826	92.4
Yes		313	7.6
Unknown^a^		1	0.0
Hypertension			
No		2654	64.1
Yes		1446	34.9
Unknown^a^		40	1.0
Hyperlipidaemia			
No		3263	78.8
Yes		791	19.1
Unknown^a^		86	2.1
Angina			
No		3949	95.4
Yes		171	4.1
Unknown^a^		20	0.5
Myocardial infarction			
No		3984	96.2
Yes		149	3.6
Unknown^a^		7	0.2
Coronary artery bypass graft			
No		4084	98.6
Yes		52	1.3
Unknown^a^		4	0.1
Percutaneous coronary intervention			
No		4031	97.4
Yes		105	2.5
Unknown^a^		4	0.1
Other heart disease (e.g. valve disease)			
No		3914	94.5
Yes		216	5.2
Unknown^a^		10	0.2
Stroke/TIA			
No		4042	97.6
Yes		92	2.2
Unknown^a^		6	0.1

*ARAD* Australian Rheumatology Association Database, *AS* ankylosing spondylitis, *PsA* psoriatic arthritis, RA rheumatoid arthritis, *TIA* transient ischaemic attack

^a^Participant reports that they do not know, or are unsure

^b^Range 0–3 where a higher score indicates greater disability

Table 2 presents disease-modifying anti-rheumatic drug (DMARD) use at the time of enrolment in the ARAD: the majority of participants were recruited on current anti-TNF biologic therapy (56.8%), with some on alternative biologics (3.1%), and 36.8% of participants were biologic-naïve at ARAD enrolment. At baseline, 1776 (56.3%) participants with RA, 265 (64.5%) participants with PsA and 437 (78.0%) participants with AS were taking a biologic therapy. Current methotrexate use was reported by 55.6% of participants at enrolment, 39.0% were currently taking prednisone or prednisolone and 51.4% were currently taking NSAIDs.

**Table 2:** DMARD usage at ARAD enrolment (*n* = 4140)

	Number	Percentage
Biologic use		
Never taken	1525	36.8
Currently taking anti-TNF biologics	2350	56.8
Currently taking other biologics	128	3.1
Abatacept	32	0.8
Anakinra	7	0.2
Rituximab	66	1.6
Tocilizumab	23	0.6
Previous use	121	2.9
Unknown^a^	16	0.4
Methotrexate status		
Never taken	977	23.6
Currently taking	2302	55.6
Stopped taking	856	20.7
Unknown^a^	5	0.1
Prednisone/prednisolone status		
Never taken	1529	36.9
Currently taking	1613	39.0
Stopped taking	969	23.4
Unknown^a^	29	0.7
NSAID status		
Not currently taking	2011	48.6
Currently taking	2129	51.4

*ARAD* Australian Rheumatology Association Database, *DMARD* disease-modifying anti-rheumatic drug, *TNF* tumour necrosis factor, *NSAID* non-steroidal anti-inflammatory drug

^a^Participant reports that they do not know, or are unsure of the answer

The study period comprised a total of 19,627 patient-years. Therapy was primarily anti-TNF (12,555 patient-years, 64.0%) or other biologics (1963 patient-years, 10.1%), while 10.0% (1955 patient-years) had ceased biologic therapy and 15.9% (3116 patient-years) were biologic-naïve. Only 29 patient-years (0.1%) included unknown DMARD therapy. Across the study period, 552 participants (13.3%) experienced a composite cardiac event and 10 died secondary to cardiovascular causes, with only one of these 10 participants reporting a CVE during the study period before dying of a cardiovascular cause.

Univariate Cox proportional hazards regression analyses for the whole group showed that increased age, male gender, RA diagnosis, disease duration, greater disability (higher HAQ), ever smoking regularly, ever using methotrexate, current prednisone/prednisolone or NSAIDs, or a medical history of hypertension, hyperlipidaemia and diabetes were all significant predictors of CVEs at the 0.25 level of significance (Table 3). Use of biologic therapy, past but not current use of prednisone/prednisolone and any level of alcohol use were inversely associated with CVEs. Continuous variables of age and disease duration were evaluated for linearity, and there was no evidence of multi-collinearity.

**Table 3:** Unadjusted univariate Cox proportional hazards regression for factors predicting cardiovascular events in patients with inflammatory arthritis (*n* = 4140)

Factor	HR	95% CI	*p* value
Increased age (years)	1.05	1.06–1.07	< 0.0001
Greater disease duration (years)	1.02	1.02–1.02	< 0.0001
Sex (males vs females)	1.44	1.33–1.55	< 0.0001
Biologic use (referent: biologic naïve)			< 0.0001
Current TNF biologics	0.63	0.58–0.70	< 0.0001
Current other biologics	0.69	0.60–0.80	< 0.0001
Stopped taking biologics	0.97	0.85–1.10	0.59
Diagnosis (referent: rheumatoid arthritis)			< 0.0001
Ankylosing spondylitis	0.61	0.53–0.70	< 0.0001
Psoriatic arthritis	0.75	0.64–0.88	0.0004
Methotrexate treatment (referent: never)			< 0.0001
Currently taking methotrexate	1.37	1.18–1.59	< 0.0001
Stopped taking methotrexate	1.56	1.34–1.82	< 0.0001
Prednisone/prednisolone treatment (referent: never)			< 0.0001
Currently taking prednisone	1.35	1.22–1.49	< 0.0001
Stopped taking prednisone	0.85	0.76–0.95	0.003
NSAID treatment vs not currently taking	1.19	1.10–1.28	< 0.0001
Smoking regularly ever	1.50	1.39–1.62	< 0.0001
Alcohol use (referent: never)			< 0.0001
Sometimes	0.64	0.59–0.69	< 0.0001
Every day	0.85	0.76–0.95	0.01
Hypertension (referent: no)			< 0.0001
Positive history for hypertension	2.21	2.04–2.41	< 0.0001
Hyperlipidaemia (referent: no)			< 0.0001
Positive history for hyperlipidaemia	2.39	2.22–2.59	< 0.0001
Diabetes (referent: no)			< 0.0001
Positive history for diabetes	1.98	1.80–2.18	< 0.0001
Higher HAQ^a^	1.83	1.74–1.92	< 0.0001

*HR* hazard ratio, *CI* confidence interval, *HAQ* Health Assessment Questionnaire Disability Score, *NSAID* non-steroidal anti-inflammatory drug, *TNF* tumour necrosis factor

^a^Range 0–3, where higher scores indicate greater functional impairment

Multivariate analysis for the whole group (Table 4) found that, following adjustment for potential confounders, compared to the biologic-naïve, the CVE risk was reduced with anti-TNF use (HR 0.85, 95% CI 0.76–0.95) as well as use of other biologic therapies (HR 0.81, 95% CI 0.70–0.95), but was not reduced when biologic use was ceased (HR 0.96, 95% CI 0.83–1.11). After adjustment, no significant difference in the CVE rate was observed between RA and PsA (HR 0.92, 95% CI 0.77–1.10) or AS (HR 1.14, 95% CI 0.96–1.36). Co-morbid hypertension, hyperlipidaemia and diabetes were all significant positive predictors of major adverse CVEs, as were increased age, male sex, ever smoking regularly, greater disability (higher HAQ) and current treatment with methotrexate or current use of NSAIDs. Alcohol use was associated with a decreased risk of CVEs. After adjusting for other variables, disease duration was not a significant predictor of major adverse CVEs.

**Table 4:** Multivariate Cox proportional hazards regression for factors predicting cardiovascular events in patients with inflammatory arthritis (*n* = 4140)

Factor	HR	95% CI	*p* value
Increased age (years)	1.05	1.05–1.06	< 0.0001
Sex (males vs females)	1.72	1.57–1.88	< 0.0001
Biologic use (referent: biologic naïve)			0.006
Current TNF biologics	0.85	0.76–0.95	
Current other biologics	0.81	0.70–0.95	
Stopped taking biologics	0.96	0.83–1.11	
Diagnosis (referent: rheumatoid arthritis)			0.18
Ankylosing spondylitis	1.14	0.96–1.36	
Psoriatic arthritis	0.92	0.77–1.10	
Methotrexate treatment (referent: never)			0.0001
Currently taking methotrexate	1.08	0.90–1.29	
Stopped taking methotrexate	1.28	1.07–1.53	
Prednisone/prednisolone treatment (referent: never)			0.02
Currently taking prednisone/prednisolone	0.96	0.85–1.08	
Stopped taking prednisone/prednisolone	0.86	0.76–0.97	
NSAID treatment vs not currently taking	1.22	1.13–1.32	< 0.0001
Smoking regularly ever	1.17	1.07–1.27	0.0003
Alcohol use (referent: never)			< 0.0001
Sometimes	0.77	0.70–0.84	
Everyday	0.77	0.68–0.87	
Hypertension (referent: no)			< 0.0001
Positive history for hypertension	1.27	1.16–1.39	
Hyperlipidaemia (referent: no)			< 0.0001
Positive history for hyperlipidaemia	1.65	1.52–1.80	
Diabetes (referent: no)			< 0.0001
Positive history for diabetes	1.28	1.16–1.42	
Higher HAQ^a^	1.48	1.40–1.57	< 0.0001

*HR* hazard ratio, *CI* confidence interval, *HAQ* Health Assessment Questionnaire Disability Score, *NSAID* non-steroidal anti-inflammatory drug, *TNF* tumour necrosis factor

^a^Range 0–3, where higher scores indicate greater functional impairment

## Discussion

This study has demonstrated a reduction in CVEs associated with biologic use for both anti-TNF and other biologic agents in ARAD participants with RA, PsA or AS, compared with ARAD participants who were biologic-naïve. However, this protective effect for the CVE rate was not observed in those who had ceased using biologic agents. Previous studies have shown that people with any inflammatory arthritis have increased rates of both cardiovascular morbidity and cardiovascular mortality compared to the general population [4, 34–36]. However, there are few primary studies directly comparing event rates between different forms of inflammatory arthritis.

Our study explored the relationship between anti-TNF use and CVEs in the Australian context and was also able to examine three different types of inflammatory arthritis in the same cohort. A reduced risk of myocardial infarction for RA patients treated with anti-TNF agents compared with conventional DMARDs was also reported in a recently updated analysis of the British Society for Rheumatology Biologic Register (BSRBR-RA) [37]. The baseline characteristics of patients entered in the ARAD are similar to the biologic-exposed population in the BSRBR-RA [20]. A similar reduction in acute coronary syndrome events for patients with RA using anti-TNF therapy was also found in a recent Swedish cohort study [38]. A recent systematic review reported a decreased risk of CVEs in patients with RA treated with TNF inhibitors or with methotrexate, and an increased risk in those using glucocorticoids or NSAIDs [39]. This review also reported that treatment with systemic therapy decreased the risk of CVEs in patients with PsA or psoriasis. However, there was insufficient data to compare the CVE risk between individual therapies.

In the multivariate model, while current methotrexate use was not associated with any difference in CVE risk, those who had ceased methotrexate had an increased risk of CVEs compared to those who had never taken the medication. Conversely, participants who had ceased taking prednisone or prednisolone were at lower risk of CVEs compared with those who had never taken prednisone. The reasons for these associations are unclear, but there may be confounding by indication for these medications. There may also be confounders which are not accounted for, including socioeconomic factors which may influence the prescription of different therapies, or some associations may have occurred by chance.

Strengths of this study include the large database of prospective longitudinal data, which was fully utilised with the counting process method of survival analysis which counts multiple events, and contrasts with the time-to-first-event analyses which has been used in previous studies [17, 35, 40]. Our study also had a moderate mean follow-up time of 5 years, and made a direct comparison between several forms of inflammatory arthritis. Additionally, the continual reporting of participant biologic use at each questionnaire significantly reduced the potential for misclassification bias.

This article also has some limitations due to the type of study and the structure of the database. This is an observational cohort study with the choice of therapy being made by the rheumatologists and patients, and as such is only able to show an association and not causation. Therefore, there are two possible explanations for the reduction in the CVE rate. There may be an intrinsic causative benefit of biologic therapy theoretically due to anti-inflammatory properties. Alternatively, it may be due to selection bias or bias by indication: rheumatologists choose to prescribe biologics for healthier patients, or to patients with higher levels of education or socioeconomic status who are consequently at lower risk of cardiovascular disease. While it is possible that patients with higher levels of co-morbidities may not have been offered biologics given higher thresholds for general health before treatment, patients that qualify for subsidy under the Australian Pharmaceutical Benefits Scheme must have more severe or resistant disease—overall, the net direction of any bias is therefore unclear [22]. Furthermore, disease activity measures such as active joint counts or inflammatory markers are not collected in the ARAD. It was therefore not possible to account for disease activity in this analysis and it is possible that it is tighter disease control achieved by biologic therapy which led to a reduction of CVEs, rather than an intrinsic effect of the biologics themselves acting on vascular inflammation.

There were only 10 deaths from cardiovascular causes observed in our study, which is lower than that which might be expected from the general Australian population. The rate of cardiovascular death in Australia in 2015 was 151 per 100,000 persons [41]. Therefore, approximately 30 deaths might have been expected in our study which included a total of 19,627 patient-years of follow-up. This low mortality rate may reflect a recruitment bias in the ARAD—most participants are Caucasian and speak English as their first language, and approximately one third have a tertiary-level education. Higher socioeconomic status and education levels are associated with reduced risk of cardiovascular death. The results may not be generalisable to the broader population with these conditions.

Apart from cardiovascular death, in this study CVEs were identified through participant self-report and it is possible that there was under-reporting of events. It is not possible to directly compare the incidence of CVEs in our study with data for the general population in Australia due to differences in the definitions of CVEs used in the Australian Institute of Health and Welfare (AIHW) analysis of the AIHW National Hospital Morbidity Database and AIHW National Mortality Database [41].

The majority of ARAD participants have RA. The ARAD was founded in 2001 for the purpose of monitoring the benefits and safety of new therapies, particularly biologics. At that time in Australia, biologics were only subsidised by the Pharmaceutical Benefits Scheme for RA, so these patients made up the bulk of initial recruitment until biologics were subsidised for AS in 2004 and PsA in 2006 [22]. However, this should not materially affect the analysis, as comparison is between biologic therapies and biologic-naïve patients. We did not find any difference in the CVE risk between RA, PsA and AS, after adjustment for other risk factors. However, as the number of participants with PsA and AS is small relative to the number of RA participants, a false negative finding is possible. The prevalence of biologics use in the ARAD population is higher than would be expected for the Australian population of patients with these rheumatic diseases. This likely reflects recruitment bias as patients commencing biologic therapy were targeted in the early recruitment process.

Furthermore, we used a composite measure for biologic use due to the small numbers of patients treated with each individual agent. It was therefore not possible to ascertain whether there was any difference in the CVE risk between individual biologic therapies. Although the ARAD collects information on reasons for biologic cessation, it was difficult to isolate a single cause to explain the finding that the CVE risk in the group who had ceased using biologic agents was not significantly different from the biologic-naïve group. This could be because those who had ceased biologic therapy were generally resistant to biologic therapy and thus did not derive any improvement in either disease status or the CVE rate, or it could be because any protective effect from biologic use is not sustained after biologic cessation and participants returned to their previous level of cardiovascular risk. Medication use was self-reported and dosages of glucocorticoid and DMARDs were not collected. Furthermore, some participants reported they were unsure if they had certain medical conditions, or had taken some medications. However, this made up only a small proportion of data points, and is unlikely to have affected the overall results.

## Conclusions

Current use of biologics, whether anti-TNF or another mechanism of action, is associated with a reduction in the CVE rate compared to the rate among people with inflammatory arthritis who are biologic-naïve. This event reduction was no longer observed in those who had ceased biologic use. There was no difference in the CVE risk between RA, PsA and AS. These findings support the hypothesis that control of systemic inflammation in these conditions may reduce the cardiovascular risk.

## Abbreviations

ARAD: Australian Rheumatology Association Database
AS: Ankylosing spondylitis
BSRBR-RA: British Society for Rheumatology Biologic Register
CI: Confidence interval
CVE: Cardiovascular event
DMARD: Disease-modifying anti-rheumatic drug
HAQ: Health Assessment Questionnaire Disability Score
HR: Hazard ratio
ICD-10: International Statistical Classification of Diseases and Related Health Problems, 10th revision
IQR: Interquartile range
NSAID: Non-steroidal anti-inflammatory drug
PsA: Psoriatic arthritis
RA: Rheumatoid arthritis
TNF: Tumour necrosis factor
